# Nonsurgical management of an extensive spontaneous spinal epidural hematoma causing quadriplegia and respiratory distress in a choledocholithiasis patient: A case report: Erratum

**DOI:** 10.1097/MD.0000000000009729

**Published:** 2018-01-26

In the article, “Nonsurgical management of an extensive spontaneous spinal epidural hematoma causing quadriplegia and respiratory distress in a choledocholithiasis patient: A case report”,^[1]^ which appeared in Volume 96, Issue 51 of *Medicine,* Dr. Fahad Abduljabbar's second affiliation was not included and should be “Department of Orthopedic Surgery, King Abdulaziz University, Jeddah, Saudi Arabia.”
